# Management of cutaneous metastases using electrochemotherapy

**DOI:** 10.3109/0284186X.2011.573626

**Published:** 2011-05-16

**Authors:** Louise Wichmann Matthiessen, Richard Ling Chalmers, David Christopher George Sainsbury, Sivakumar Veeramani, Gareth Kessell, Alison Claire Humphreys, Jane Elisabeth Bond, Tobian Muir, Julie Gehl

**Affiliations:** 1 Center for Experimental Drug and Gene Electrotransfer (C*EDGE), Department of Oncology, Copenhagen University Hospital Herlev, Denmark; 2 Department of Reconstructive Plastic Surgery, James Cook University Hospital, Middlesbrough, UK; 3 Department of Anaesthesia, James Cook University Hospital, Middlesbrough, UK; 4 Department of Oncology, James Cook University Hospital, Middlesbrough, UK

## Abstract

*Background*. Cutaneous metastases may cause considerable discomfort as a consequence of ulceration, oozing, bleeding and pain. Electrochemotherapy has proven to be highly effective in the treatment of cutaneous metastases. Electrochemotherapy utilises pulses of electricity to increase the permeability of the cell membrane and thereby augment the effect of chemotherapy. For the drug bleomycin, the effect is enhanced several hundred-fold, enabling once-only treatment. The primary endpoint of this study is to evaluate the efficacy of electrochemotherapy as a palliative treatment. *Methods*. This phase II study is a collaboration between two centres, one in Denmark and the other in the UK. Patients with cutaneous metastases of any histology were included. Bleomycin was administered intratumourally or intravenously followed by application of electric pulses to the tumour site. *Results*. Fifty-two patients were included. Complete and partial response rate was 68% and 18%, respectively, for cutaneous metastases <3 cm and 8% and 23%, respectively, for cutaneous metastases >3 cm. Treatment was well-tolerated by patients, including the elderly, and no serious adverse events were observed. *Conclusions*. ECT is an efficient and safe treatment and clinicians should not hesitate to use it even in the elderly.

A cutaneous metastasis can be defined as “a neoplas-tic lesion arising from another neoplasm with which there is no longer continuity” [1]. Cutaneous metastases account for 0.7% to 9% of all metastases [2]. Breast cancer accounts for 51 % of the total cases of cutaneous metastases, while malignant melanoma accounts for 18% [3].

The management of cutaneous metastases often presents a challenge for the clinician as they may be widespread and may recur after radiotherapy or chemotherapy. In some cases, patients may have stable disease in sites other than the skin, and clinicians may be reluctant to use systemic chemotherapy for the skin metastases alone.

Electrochemotherapy (ECT) is a rapidly emerging and effective treatment option for cutaneous metastases from malignant tumours [4-6]. ECT uses local application of short duration, electric pulses directly to the tumour cells via an electrode, causing destabilisation of the cell membrane and thereby making it transiently permeable (electropermeabili-sation - Figure 1) [7]. Bleomycin, a chemotherapeu-tic agent used in the treatment of cancer, is under normal conditions unable to freely diffuse through the plasma membrane. However, electropermeabili-sation allows this otherwise poorly permeating agent to enter the cell cytosol, thereby greatly increasing its concentration within the tumour cell (Figure 2). In high concentrations, such as those achieved with ECT, bleomycin can cause cell death within a few minutes [8]. Preclinical studies have demonstrated a 300- to 700- fold increase in bleomycin cytotoxicity using this method of drug delivery [9-11].

**Figure 1:**
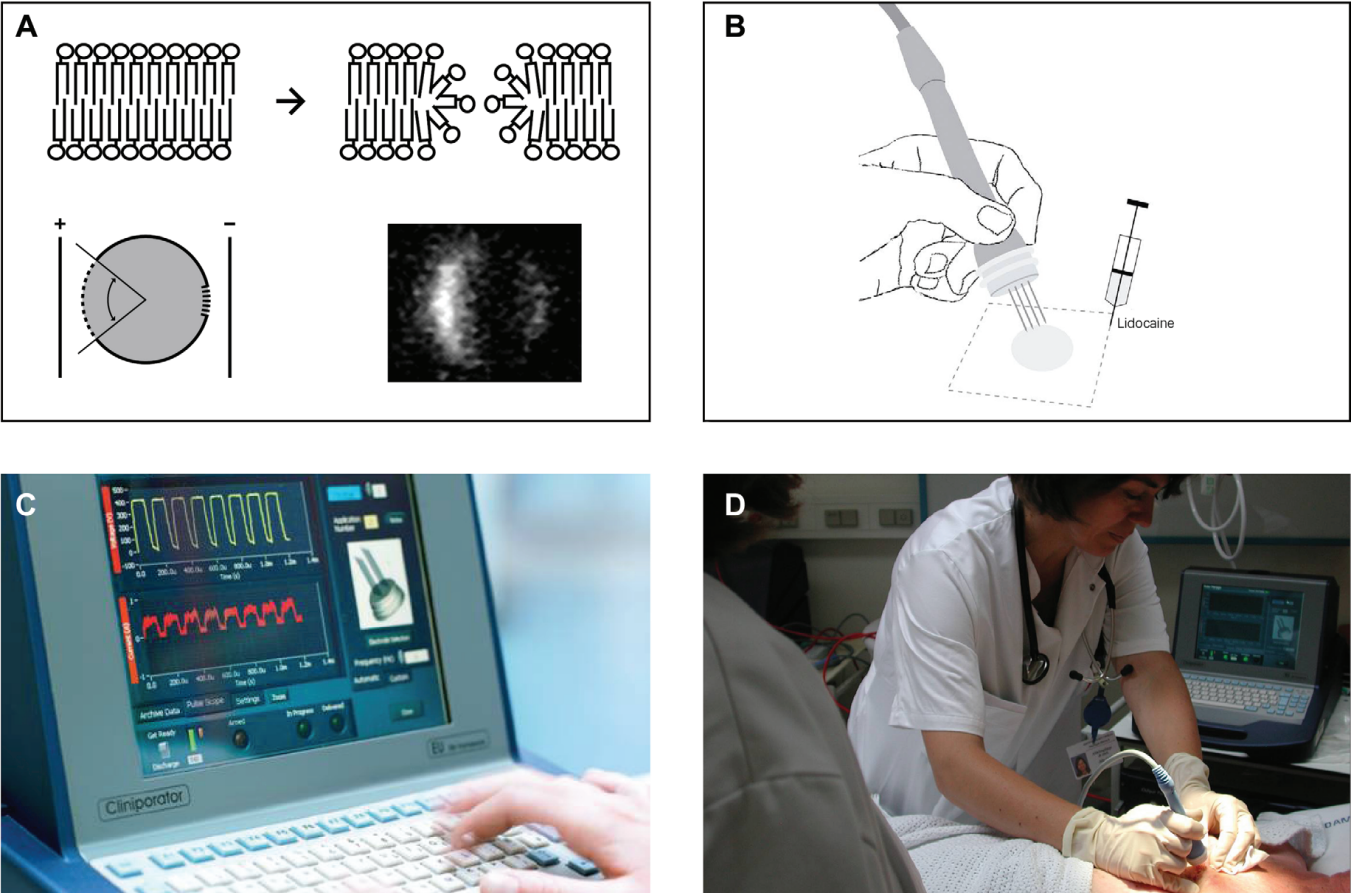
The electroporation procedure: A. Electroporation occurs when an applied external field exceeds the capacity of the cell membrane. The formation of permeable areas happens in the frame of less than a second whereas resealing happens over minutes. As the resting transmembrane potential is negative on the inside respective to the outside, the first part of the membrane that will be permeabilised is the pole facing the positive electrode. The positive electrode should be imagined in the left of the picture and the negative electrode on the right. Pulses were delivered to a cell suspended in medium containing propidium iodide and after the pulses propidium iodide is trapped within the cells [9]. B. The application of pulses to skin tumours must be preceded by local or general anaesthesia, in local anaesthesia the lidocain is injected around the metastasis. C. The cliniporator equipment allows monitoring of voltage and current during the pulse. D. A treatment situation is shown where a patient is receiving local injection of bleomycin followed by application of pulses under local anaesthesia. The application of pulses lasts only a few minutes in total.

**Figure 2:**
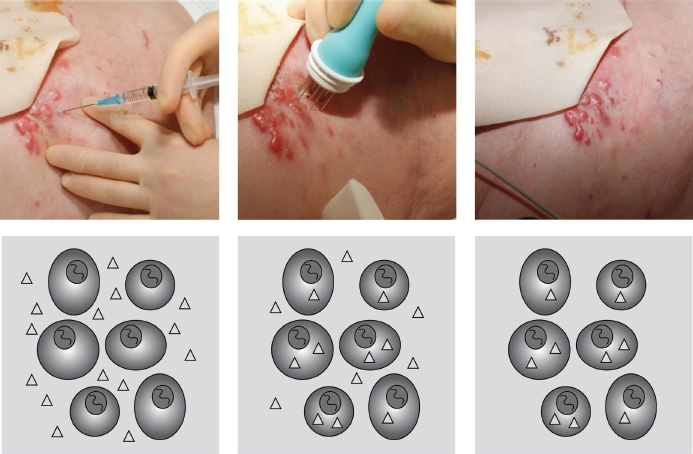
Electrochemotherapy. In the right panel, bleomycin is injected at the tumour site, at a concentration of 1000 IU/ml (1 U/ml). In the middle panel the electric pulses are subsequently applied, cells are permeabilised and the drug enters. In the left panel the cells reseal after a few minutes and the extracellular drug is washed out while the internalised molecules remain trapped intracellularly.

ECT was originally employed for treatment of metastatic head and neck cancer [12] and has since been used in the treatment of cutaneous metastases from tumours independent of histology [5,13,14]. ECT can be used where surgery is not an option and is also efficient in chemotherapy-resistant and radiotherapy-resistant lesions [5,15]. Treatment may provide palliation particularly where there is pain or bleeding from cutaneous metastases [16]. It is well tolerated with few side-effects, allows for immediate recovery and can be repeated [4,5]. In 2006 a European study was published (the ESOPE study) [5] and with that the standard operating procedures [17] which describe the ECT procedures in detail.

The present study aims at continuing the exploration of ECT as a highly effective treatment in order to improve and evaluate its benefits. To this end, we created the International Network for Sharing Practice in Elec-troChemoTherapy (INSPECT) database, the purpose of which is to gather, share and publish clinical data and experience. This is the first report from this network.

## Patients and methods

### Study design

Patients were recruited consecutively at two institutions: Copenhagen University Hospital Herlev, Denmark and James Cook University Hospital, Middlesbrough, UK. The primary endpoint was response rate. Secondary endpoints included safety and response rate according to size.

Patients at Herlev Hospital with cutaneous metastases, for whom no further surgery or conventional treatment was feasible, could be offered treatment with ECT within the framework of a non-randomised phase II study. Approval was granted by the local ethics committee and the Danish Medicines Agency.

Approval in the UK was granted in 2007 by the clinical effectiveness subcommittee for treatment of metastatic skin and subcutaneous lesions, palliation of bleeding or painful lesions and primary treatment of cancers not amenable to surgical excision or conventional treatments.

Patients fulfilling the inclusion criteria were sequentially enrolled and all patients signed informed consent before inclusion.

Patients eligible for inclusion had histologically proven malignant cancer with measurable cutaneous or subcutaneous tumour nodules suitable for application of electric pulses. Patients had been offered standard treatment options, were ≥18 years old, had ECOG performance status ≤2, had an expected life expectancy of at least three months and, where appropriate, were using adequate contraception. A platelet count ≥50 mia/l was required, with a prothrombin time ≤40 s and an activated partial throm-boplastin time in the normal range.

Patients were ineligible if they had previously had allergic reactions to bleomycin or to any of the components required for anaesthesia, if the cumulative dose of 250 mg bleomycin/m^2^ (400.000 IU bleo-mycin/m^2^) had previously been exceeded, had chronic renal dysfunction (serum creatinine >150 μmol/l) or acute lung infection.

Follow-up was planned for up to six months. Patients who had been started on systemic antineo-plastic treatment after ECT were excluded from the study at that time.

### Procedure

The ECT sessions were performed based on the standard operating procedures for electrochemotherapy [17]. Bleomycin was administrated either intratumourally (i.t.) or intravenously (i.v). The decision to treat either i.t. or i.v. was based on the number of cutaneous metastases to be treated and the size of the metastases.

General anaesthesia, was preferred for multiple metastases, large metastases (>3 cm), metastases adhering to the periosteum or situated in sensitive regions (e.g. face and scalp), and in accordance with patient preference.

### Injection of bleomycin

*Intratumoural treatment (for small or few cutaneous metastases)*. Bleomycin was injected into the cutaneous metastases according to size. Pulses were delivered after administration of the drug (all pulses must be administrated within 10 minutes of bleomycin injection).

*Intravenous treatment (for large or many cutaneous metastases)*. Bleomycin was injected intravenously (15000 IU/m^2^ = 15 U/m^2^ which is approximately equal to 8.5 mg/m^2^ bleomycin depending on the activity of the drug and the manufacturer). Pulses were delivered 8-28 minutes following injection when bleomycin is known to be present in high concentration in the tumour [18,19].

### Anaesthesia

*Local anaesthesia (for small or few cutaneous metastases)*. Lidocaine with epinephrine was injected around the metastasis (Figure 1). The electrode was placed in and around the metastasis and the pulses administered. Under local anaesthesia, patients do not feel the insertion of the electrode needles but do feel a brief local muscle contraction upon administration of the electrical impulse.

*General anaesthesia (for large or many cutaneous metastases)*. Anaesthesia for electrochemotherapy (ECT) was tailored to the patient's condition, the position of the lesions, the extent of treatment and the special considerations pertaining to general anaesthesia and the use of bleomycin [20].

Depending on the clinician's choice, one of the following electrodes was used: 1) Type I electrodes: two plates with a 6 mm gap between the plates; 2) Type II electrodes: two parallel rows of needles with 4 mm between the rows; 3) Type III electrodes: a hexagonal array of electrodes with 7.9 mm between the needles.

Electric pulses (eight pulses of 100 μs duration) were delivered using a square wave electroporator (IGEA, Carpi, Italy). The applied voltage was 1.3 kV/ cm for plate electrodes and 1.0 kV/cm for needle electrodes, i.e. for the type II needle electrode with a 4 mm gap between the needles the applied voltage was 400 V. For type I and II electrodes, the pulses are applied with 1 Hz or 5 kHz, whereas for type III electrodes, pulses can only be applied with 5 kHz. Electrodes were single use. The duration of the procedure was recorded from the start of the bleomycin injection to the completed delivery of the last pulse. After ECT, the treated metastases were covered with standard dressings where necessary.

### Tumour response and safety assessment

**Figure 3:**
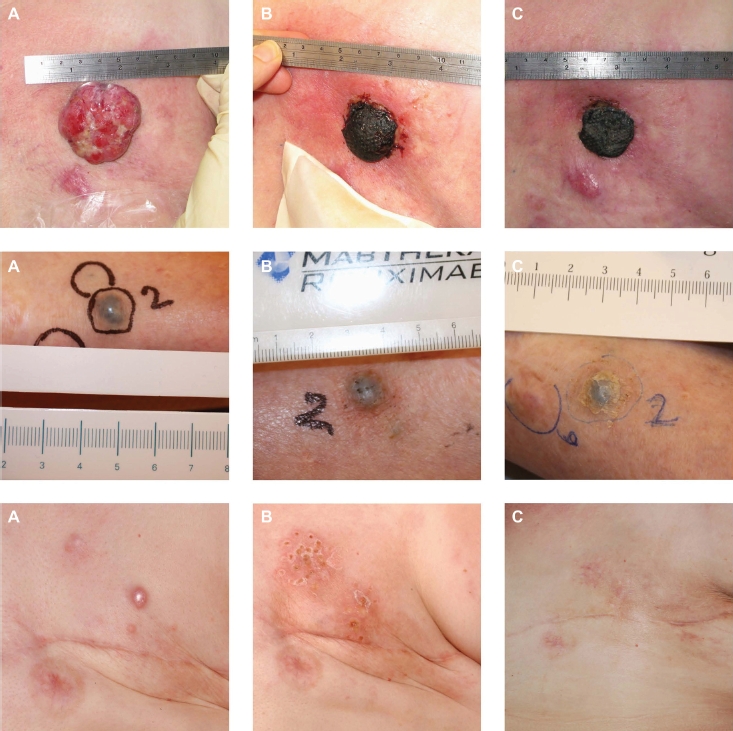
Results. Top: ECT treatment of a 75-year-old woman with metastatic breast cancer. Previously the patient underwent surgery and received endocrine therapy, chemotherapy and radiotherapy. The patient was treated with one single treatment of ECT in local anaesthesia with intratumoural injection of bleomycin. A. before treatment, B. two weeks after treatment, C. four weeks after treatment. A crust formed and after four weeks showed signs of falling of. The patient was very satisfi ed with the treatment and had no side-effects. Middle: ECT treatment of an 82-year-old woman with malignant melanoma. The patient was diagnosed in 2007, since had local spread on the lower limb and metastasis to the lungs. The patient was not suited for temozolamide, immunotherapy or isolated limb perfusion. The patient was treated with ECT in general anaesthesia and intravenous injection of bleomycin. A. before treatment, B. 24 days after treatment and C. three months after treatment. After three months the metastasis is in partial response. Bottom: ECT treatment of a 38-year-old woman with metastatic breast cancer. Previously the patient underwent mastectomy, radiotherapy and chemotherapy. The patient was treated with one single treatment of ECT in general anaesthesia with i.v. injection of bleomycin. A. before treatment, B. 10 weeks after treatment, C. two years and two months after treatment with complete resolution despite ongoing systemic disease.

Evaluation of the tumour response was by measurement of the extension or regression of the treated metastases. This was documented using digital photography. A maximum of seven cutaneous metastases per patient were registered as target lesions in order not to skew data by inclusion of patients with very large numbers of cutaneous metastases. The response was registered for each target lesion and new cutaneous metastases were not considered in response evaluation but could be treated in a second ECT session. The response rate was evaluated similarly to the Response Evaluation Criteria in Solid Tumours (RECIST version 1.0) [21]: Complete response (CR) was defined as disappearance of the target lesion; partial response (PR) with at least 30% decrease in the diameter of the target lesion; progressive disease (PD) with at least 20% increase in the diameter of the target lesion and stable disease (SD) with neither sufficient shrinkage to qualify for PR or sufficient increase to qualify for PD. In some cases with exophytic ulcerated tumours evaluation was not possible due to crust formation (Figure 3).

Safety was reported in the form of adverse events using Common Toxicity Criteria version 3.0. Patients were asked if they would potentially agree for another session as a measure of how patients felt about the treatment procedure.

### Statistical consideration

Descriptive methods were employed for statistical analysis using SPSS 13.0.

Patients were followed for six months, but excluded from further evaluation if new antineo-plastic treatment was started within the six months.

All patients treated with ECT were included for evaluation of efficacy and safety.

## Results

### Patient population

A total of 52 patients with cutaneous metastases were enrolled between June 2007 and April 2010. Table I presents patient characteristics at baseline. Fifty-one patients underwent electrochemotherapy for 196 cutaneous metastases from primarily malignant melanoma or breast cancer (Figure 3). In one patient with malignant melanoma in the head region, treatment was not given due to poor lung function.

**Table I:** Patients' characteristics at baseline.

	Patients Total (N = 52)	Patients (%)	Patients Herlev, N = 30	Patients Middlesbrough N = 22
Median age in years (range)	69.6 (38.9-94.7)		72.1 (53-89.8)	68.3 (38.9-94.7)
*Age distribution*^1^				
80 +	11	11%		
70 +	25	48%		
60 +	44	85%		
50 +	48	92%		
*Sex*				
Female	35	67%	24	13
Male	17	33%	6	8
*ECOG^2^ performance status*				
0	35	67%	58%	84%
1	12	23%	33%	5%
2	5	10%	9%	11%
*Previous Treatment*				
Surgery	42	81%	71%	95%
Radiotherapy	20	38%	42%	21%
Chemotherapy	21	40%	42%	37%
No previous treatment	8	15%	21%	0%
*Number of metastases treated pr. patient*^3^				
Median (range)	3 (1-7)		3 (1-7)	4 (1-7)
*Diagnosis*^4^				
Malignant Melanoma	21	40%	36%	47%
Breast Cancer	15	29%	33%	21%
Adenocarcinoma (other than breast)	5	10%	15%	0%
Basocellular Carcinoma	5	10%	9%	11%
Squamous Cell Carcinoma	3	6%	6%	5%
Other	3	5%	0%	16%
*Location of metastasis*				
Chest	79	40	40	41
Lower limbs	54	28	22	36
Head and Neck	30	15	22	6
Scalp	21	11	12	9
Upper limbs	6	3	4	1
Abdomen	5	3	1	5
Back	1	1	0	1
*Size of metastases*				
Median diameter in mm (range)	12 (1-200)		15 (2-200)	5 (1-140)
≤3 cm	138			
>3 cm	24			

1 Number of patients at or above a given age.

2 ECOG = Eastern Cooperative Oncology Group.

3 Maximum seven metastases per patient registered, for 51 patients.

4 No significant differences among distribution of diagnosis of primary tumour among centres could be observed (p = 0).

Forty-five patients were evaluable for safety and toxicity, and 24 patients with 97 cutaneous metastases had a follow-up of 60 days or more. Eleven patients received a second treatment with ECT.

The median diameter of the cutaneous metastases was 12 mm ranging from 1 mm to 200 mm. Locations of the cutaneous metastases are presented in Table I.

### Treatment

Treatment data are listed in Table II.

**Table II:** Treatment data and response.

TREATMENT DATA	All Patients (n = 51)	All Patients (%)	Herlev	Middlesbrough
*Chemotherapy*				
Bleomycin I.T	21	41%	41%	42%
Bleomycin I.V.	30	59%	59%	58%
*Anaesthesia*				
Local	23	45%	50%	37%
General	28	55%	50%	63%
*ECT session duration^1^, hours:minutes*				
Median (range) (hh-mm)	00:16 (00:05-01:27)		00:29 (00:08-01:27)	00:18 (00:05-00:35)
*Would agree for another session*				
yes	46	90%	87%	90%
no	4	8%	13%	5%
no answer	1	2%	0	5%

RESPONSE	All Metastases (n = 97)^2^	Metastases (%)	Herlev	Middlesbrough

*Response for registered metastases^3^*				
CR	58	60%	54%	68%
PR	18	19%	20%	17%
SD	11	11%	18%	2%
PD	7	7%	4%	12%
Not evaluable	3	3%	5%	0%
*Time from treatment to CR (days)*				
Median (range) (days	47 (16-110)		41 (16-110)	63 (38-100)
*Size of metastases S30 mm (n = 84)*				
CR	57	68%		
PR	15	18%		
SD	5	6%		
PD	5	6%		
Not evaluable	2	2%		
*Size of metastases >30 mm (n = 13)*				
CR	1	8%		
PR	3	23%		
SD	6	46%		
PD	2	15%		
Not evaluable	1	8%		

1 Data available for 42 patients, the time is from start of chemotherapy administration till the last pulse was given. This means it does not include time anaesthesia. One patient with the procedure lasting 1 hour and 9 min was treated in local anaesthesia with i.t. injection of bleomycin had three nodules where treatment of the first nodule was finished before the anaesthesia of the next nodule began. One patient with the procedure lasting 1 hour and 27 min was treated in general anaesthesia with i.t. injection of bleomycin had seven nodules where treatment of the first nodule was finished before injection of bleomycin in the next nodule. This explains why some procedures lasted longer than one would expect.

2 24 patients with 97 metastases with a follow-up > 60 days.

3 Maximum seven metastases per patient registered.

Patients treated with i.v. or i.t. administration of bleomycin had a median number of three treated cutaneous metastases. The median size of the cutaneous metastases treated with i.v. bleomycin was 10 mm (range 1-200 mm) and for i.t. 9 mm (range 1-50 mm).

### Anaesthesia

Of the 51 treatments, 28 (55%) were performed under general anaesthesia and 23 (45%) were performed under local anaesthesia (see Table III). There was no statistical difference between number of nodules per patient and choice of anaesthesia.

**Table III:** Choice of anaesthesia according to location of metastases and size.

	Local anaesthesia		General anaesthesia	
*Location of metastases*				
Chest	23	32%	56	46%
Lower limbs	31	42%	23	19%
Head and Neck	11	15%	19	15%
Scalp	4	5%	17	14%
Upper limbs	3	4%	3	2%
Abdomen	1	1%	4	3%
Back	0	0	1	1%
*Size of metastases*				
Median (range) (mm)	7.5 (1-60)		10 (1-200)	
*Number of metastases per patient*				
Median (range)	3		4	

### Choice of electrode

The electrodes used for treatment were as follows: type II electrodes for 119 (61%) of the cutaneous metastases; type III electrodes for 47 (24%); type I electrodes for 21 (11%); both type I and II electrodes for two (1%) and both type II and III electrodes for seven (4%).

### Duration of procedure

Data on duration were available for 42 procedures. The median duration of a treatment session from start of bleomycin administration to last pulse delivered was 20 min (range 5 min to 1 hour and 9 min) for local anaesthesia and 25 min (range 11 min to 1 hour 27 min) for general anaesthesia (see Table II).

### Treatment response

Six patients were lost to follow-up before evaluation due to systemic disease progression. Forty five patients with 162 treated cutaneous metastases had a median follow-up of 79 days (range 8-180), and 24 patients with 97 nodules had a follow-up >60 days. Responses are presented in Table II.

For patients with a follow-up >60 days, CR was observed in 58 (60%) metastases, PR was observed in 18 (19%) metastases, SD was observed in 11 (11%) metastases and PD in seven (7%) metastases. Response was not evaluable in three (3%) metastases.

### Safety

No serious adverse events (SAE) were observed. Reported adverse events were flu-like symptoms one to two days after treatment (five patients, 10%), pain in the treated area one to two days after treatment (five patients, 10%), ulceration of treated area (two patients, 4%), cough (one patient, 2%), allergic skin reaction (one patient, 2%) and anxiety (one patient, 2%). There was no CTC grade 3 or 4 tox-icity. Most side-effects were seen when treated under general anaesthesia with systemic administration of bleomycin.

Of the 51 patients treated, 46 (90%) would agree to another treatment, four (8%) would not agree to another treatment and one patient is not accounted for.

## Discussion

### Cutaneous metastases: A challenge in cancer treatment

Cutaneous metastases or recurrent malignant disease in the skin, particularly after treatment of malignant melanoma, head and neck carcinoma or breast cancer, is often difficult to manage. Patients have often received multimodal treatment with surgery, radiotherapy and chemotherapy and are faced with obviously progressing disease. The uncontrolled cutaneous metastases can, in many ways, adversely affect self-esteem and body image. The cutaneous metastases and the treatment of the cutaneous metastases will seldom affect life expectancy, but may be very important for the patient's quality of life.

### Electrochemotherapy

Electrochemotherapy is a method where the combination of electric pulses and bleomycin increases the cytotoxicity of bleomycin 300-700 times [9-11]. When electric pulses are delivered to tissue in the presence of bleomycin, the cell membrane becomes permeable and bleomycin enters the cell where it is trapped. The large increase in bleomycin cytotoxicity makes it possible to do “once-only” treatment suitable for the palliative patient. The clinical effectiveness of ECT was first demonstrated in head and neck squamous cell tumour nodules in 1991 [22]. Subsequent clinical investigation has shown that ECT using bleomycin is also a feasible and effective treatment for cutaneous and subcutaneous metastases of other malignancies [14].

The ESOPE study in 2006 [5] produced standard operating procedures for ECT treatment (including dosage, pulse parameters, electric pulse generators and electrodes), pain control and indications for treatment. The ESOPE study demonstrated that electrochemotherapy is an easy, highly effective and safe treatment for small (<3 cm) cutaneous or subcutaneous metastases of various malignancies. The objective response rate after one treatment was 85%. Similar results have been demonstrated by Campana et al. [13] and additionally by many case-reports and smaller studies [23-25].

### Electrochemotherapy in routine use

In the present study we have tried to manage cutaneous metastases with ECT as a routine procedure in two cancer centres. The primary endpoint of this study was response rate.

Fifty one patients from Denmark and the UK were treated for 192 cutaneous metastases with ECT - the majority with either malignant melanoma or breast cancer. This is in agreement with breast cancer being the most common malignancy with cutaneous metastases in women and malignant melanoma being the most common in men [3].

ECT treatment in this study was provided as a palliative procedure to patients with performance status <2. A broad spectrum of patients was included, which is reflected in six patients lost to follow-up before any evaluation and only 24 patients having a follow-up >60 days. Some patients travelled long distances to reach the centre offering ECT which hindered follow-up, and some patients had systemic progression during follow-up and were offered other antineoplastic treatment. These factors may explain the relative high rate of patients lost to follow-up before any evaluation and only 47% of patients having a follow-up period >60 days.

The classic RECIST criteria [21] were unsuitable as tumour assessment in evaluation of ECT treatment as RECIST includes measurable lesions in other organs if present, a minimum size of 1 cm and a maximum five lesions per organ. Also the aim of ECT is local and not systemic control. Instead, the definitions of CR, PR, PD and SD from RECIST were adapted and seven cutaneous metastases were registered as target lesions. This seems a feasible way to evaluate ECT.

In this study, objective response rate (OR) for patients with a follow-up period of >60 days was 86% for cutaneous metastases <3 cm and 31% for cutaneous metastases >3 cm. The metastases were divided into smaller or larger than 3 cm to enable comparison with previous studies. The response rate for the cutaneous metastases <3 cm is similar to the ESOPE [5] and other studies [13,23,24], whereas for larger metastases, the OR is considerably lower, which is in agreement with previous observations [13]. In patients with large volume disease, the purpose is not necessarily to eradicate the cutaneous metastases, but to obtain palliative relief in terms of decreased odour, exudate and bleeding. Therefore, SD can be the aim of ECT treatment for large volume disease. However, in the management of small cutaneous metastases, control and disappearance of cutaneous metastases can be the aim of treatment. Due to the low incidence of complications, treatment can be repeated several times in order maintain local control or obtain control if not achieved by the first treatment. In this study 11 of the 51 patients were resubmitted for treatment, either due to new metastases or progression of previously treated metastases, with no SAE's observed.

### ECT and its place in oncological practice

For patients with cutaneous metastases, local control during their remaining life period is the goal of treatment. Regional and local techniques such as palliative surgery, re-irradiation, hyperthermia, isolated limb per-fusion and isolated limb infusion can be offered to patients with cutaneous metastases in order to provide local symptom control. When offering treatment to patients, the risk of complications and toxicity should always be carefully addressed and the likely benefit should always be compared with the risks to the patient. Electrochemotherapy offers a minimally invasive local treatment with swift symptomatic relief and few side-effects. ECT can also, as shown in this study with 48% of patients being >70 years, be offered to elderly patients for whom other treatments may not be a possibility.

In this study, treatment was performed at two different centres - a department of plastic surgery and a department of oncology - with similar results. This demonstrates that the treatment functions well in different types of units. ECT may easily be implemented as limited training is needed.

## Conclusion

In conclusion, our results in concordance with previous studies suggest ECT is an efficient treatment that may improve quality of life in patients with meta-static disease and clinicians should not hesitate to use it even for elderly patients. ECT is simple to administer, and can therefore be implemented by smaller hospital units with resultant benefits for patients.

In our two centres, we concurrently found ECT to be an excellent treatment choice for the patient suffering from cutaneous metastasis where other treatments have failed.

We would recommend that more centres offer ECT and that referral for this once-only and simple treatment should be considered.
